# Nervous Energy

**Published:** 1885-10

**Authors:** E. Parsons

**Affiliations:** Savannah, Georgia.


AMERICAN JOURNAL

OF

DENTAL SCIENCE

VOL. XIX. THIRD SERIES.—OCTOBER 1885. No. 6.

ARTICLE I.

### NERVOUS ENERGY.

BY DR. E. PARSONS, SAVANNAH, GEORGIA.

[Read before the Georgia State Dental Society, May, 1885.]

GENTLEMEN—The subject I have chosen for your consideration at this, our Annual Meeting, is "Nervous Energy, how Actuated, and its Varied Phenomena." No one can question the importance of knowing all that can be known about it.

There is an invariable law by which means mind acts on matter, and it is my purpose, in this paper, to briefly elucidate what I have learned by reading, observation and experience on the subject. The great advantage of meeting in council is an increase in knowledge on all subjects in any way relating to our profession. We have many things yet to learn that will be, when known, of great benefit to both ourselves and the public.

Science demands a full and free investigatinn of all or any causative principle by which life is manifested, or death produced. So long as we draw our conclusions only from appearances, we shall often be deceived in a correct diagnosis; consequently often fail to cure diseases that come within the legitimate bounds of our specialty.

Proper remuneration for our services are absolutely necessary for the respectable maintainance of ourselves and those dependent on us; but our best men are laboring unweariedly in their endeavor to elevate our standard throughout the world, but particularly in our own country, and this Society can do much to help them in their onward march, developing all possible improvements in Scientific Dentistry.

Again, we all have a full consciousness of three things —we love, we think, we act. But few have a scientific knowledge of the means employed by which mind acts on matter. There are such varied forms and circumstances controlling its development, that we need not marvel at anything that comes within the sphere of our observation. As we are brought face to face with almost every possible condition of the nervous system, our opportunities for investigating the various manifestations of nervous energy, its source and supply, may we not equal any other specialty in solving the great problem of cause and effect manifesting nervous energy? As the brain is the seat of all sensation, I briefly present some of the best authenticated views of its organization. I think you all will agree with me that it is wonderfully constructed by Infinite Wisdom for the development of the finite mind. In the elucidation of my subject, let us not forget the fact that the blood has much to do with the various conditions of the nervous system. It holds, or should contain in solution, all the elements necessary for the growth and sustenance of every organ in the body; it is both a receiver and a giver; it is fed from what we eat and drink, without which it cannot perform the office intended.

### THE BRAIN.

The brain is divided by the septum into two lobes, right and left sides; also, into the cerebrum and the cerebellum, front and back sides. Phrenologists divide the lobes into about forty convolutions, assign to each a distinct office, and by careful observation of each as to their development, profess to know individual character, and point out what kind of occupation one, by nature, is best fitted for.

In 1840, Dr. Sherwood, of New York, by ingenious experiments, demonstrated the fact that the brain has four large poles, two in the cerebrum and two in the cerebellum, and from these proceed not only the convolutions, but every nerve in the body. By these, and experiments in animal magnetism, he maintained that animal magnetism is the motive power of the human system, and without it there can be no connection between mind and matter.

In the Fall of 1844, I invited several of our most eminent physicians to meet me at my office to witness some experiments in magnetism. My subject for demonstration was a youn man of unimpeachable character, twenty-three years of age. My visitors were very skeptical on the subject of magnetism There had been some public exhibitions, but the result did not satisfy them.

I said, as the science of phrenology is ridiculed by some, I wished first to exhibit each convolution of the brain, in a state of exaltation, while he is as wide awake as we are. To prevent any suspicion of collusion, I handed them a chart containing the names of the different convolutions of the brain, and requested them to write on paper any question or the name of the organ, for me to excite.

The first paper had on it mirth. I placed the point of my finger over the organ, and he immediately broke out into an uncontrollable fit of laughter. I passed off the influence and he instantly became calm. They asked him what he laughed at. He said he did not know; he could not help it.

The second paper had on it veneration. I excited the organ, and he immediately bowed his head and assumed the attitude of the most zealous pietist, and appeared to be in earnest prayer.

The next paper had on it music. I excited the organ, and he immediately commenced singing with as much earnestness as if his life depended on it.

The next paper had on it combativeness. I excited the organ; he immediately doubled his fist and pitched into an imaginary enemy in the most vigorous manner possible.

Not to take up too much space in this paper, I will only add, we went through with about twenty of the organs with equally marked results, which both pleased and astonished my friends. They said they now thought there was much more truth in the science of phrenology than they had supposed possible. I then said you have seen the effect of so-called animal magnetism; I will now exhibit a different phase of it. I magnetized him in the usual way, and said he is now as oblivious to all external impressions as if his five senses had no existence. Examine him and satisfy yourselves.

After a thorough examination, Dr. Richardson said he believed he could cut off his leg and he would not feel it. I demagnetized him, which restored him to full consciousness. They questioned him about it. He said he did not remember anything done in that state. They then said if it was practicable it would be a good thing in surgical operations. They thanked me for the pleasure of witnessing the experiments, and retired.

To understand the different nervous conditions of patients is of vast importance to both dentists and physicians. This cannot be attained without close observation and experience. If the nervous temperament of a patient is known, we shall have a key to guide us in our treatment in every individual case. Temperament is usually divided into six distinct classes:

1st. Nervous bilious; 2d. Nervous sanguine; 3d. Nervous lymphatic; 4th. Bilious nervous: 5th. Sanguine nervous; 6th. Lymphatic nervous.

You have doubtless observed a difference in the quality of human teeth. We usually find the best in the nervous bilious temperament, and the poorest in the lymphatic nervous. Viciated tastes and habits do not change the shape of the teeth when once formed, but their quality. Science demands discrimination under varied circumstances; it is not possible to treat all alike, and be equally successful. I shall refer to this again below.

I now present you with a few incidents in practice which may serve as a basis for a better elucidation of my subject:

1st. In the Fall of 1835, I was called to see a gentleman at eight P. M., represented to be suffering greatly, and unable to come to my office. I was introduced to a large man walking the floor in great agony. Seating him in a chair, I found the left side of the face swollen; a purple colored spot over the antrum; the first molar on the left upper jaw filled with gold; tooth firm, but evidently devitalized. I diagnosed the trouble to be abscess in the antrum. I extracted the tooth; he sprung out of the chair and dropped on the floor, face downwards, and quivered like an ox struck on the head with an ax. I used cold water freely to his head, and soon brought him to; placed him back in the chair; made a free passage through the front labial socket into the antrum, and the pus flowed freely. He then laid down on the bed much relieved; gave him a half grain of opium; waited about twenty minutes and injected the antrum with warm green tea; directed his head to be kept cool with cloths wet with cold water; left him with a promise to call again in a few hours; called again about three P. M.; was told he had been sleeping several hours; I injected the antrum with a weak solution of nitrate of silver; saw him the next morning and again injected the antrum with a much stronger solution of nitrate of silver; the swollen cheek appeared almost natural; said if he needed my service any more to come to my office. About a week later he called on me; brought me a sack; said a few hours before that he blowed it out through the left nostril; it was about one and a fourth-inch long, and about the size of an ordinary goose-quill; it was soft with a leathery like appearance; said he was all right; paid his bill; have not seen him since.

2d. In September, 1836, I was called by a physician to see his wife. He said she was suffering terribly from facial neuralgia, and thought it was caused by a tooth. She was in her eighth month of pregnancy. He had applied the usual remedies, which gave no relief. I examined her teeth; found both the third molars on upper jaw decayed, and on slightly tapping them with the handle of an instrument, the pain was greatly increased. He said he was afraid of the consequences, in her state, of having the teeth extracted. I told him nothing else would give relief. She said take them out, it cannot be worse than I am now suffering. I parted the gums from the teeth with a lance; I had barely completed this part of the operation when she fainted; her mouth was open; I took my forceps and extracted both teeth; brought her head forward to prevent the blood running down her throat; we soon brought her back to consciousness, and the first thing she said was: "I cannot have them out." Her husband said: "Darling, they are both out." She said, "Are they? I did not feel it; I am so glad; the pain is all gone." The Doctor said to me—"You are a bold man: I would have stopped you if I could, but you was too quick for me." I afterwards learned that no serious result followed the operation.

3d. In August, 1856, a young lady, aged about twenty, came to my office at eight o'clock A. M. Temperament nervous, lymphatic. Said she had not slept a wink all night. Her face was pale, hands cold, pulse feeble. Said she had a mortal dread of having a tooth extracted. I put my mouth-mirror into her mouth for examination, and saw the tooth caused the trouble. In an instant she fainted. I took my forceps and extracted the tooth, used restoratives and soon brought her to. The first thing she said was—"I cannot have it out." I showed her the tooth. She said: "Oh! I am so glad I did not feel it;" and left the office laughing about it.

4th. In the Fall of 1858 a lady called to have the two upper front incisors filled. She appeared to be middle-aged, and apparently in good health. On examination, the teeth were very close together—not badly decayed, but must be separated for sufficient room to enable me to do the work properly. Our only means, then, was either wedging or filing them. I filed about one quarter of what was necessary, and she fainted. I then filed as rapidly as possible while she was unconscious, and completed this part of the operation, and used restoratives, and soon brought her to. I gave her a glass of wine, and completed the operation without further trouble.

5th. In the Fall of 1874, a lady called to have a tooth extracted. She appeared to be in good health; said she was almost distracted with toothache; was afraid to take chloroform; was afraid as of death without it. I said the pain would be only momentary, and would not kill her. I extracted the tooth, and she fainted. My usual remedy in such cases was hartshorn and cold water. Through mistake, I took up a vial of the Essence of Gaultheria, poured a little on a handkerchief, held it to her nose, and was surprised to see how quickly she recovered consciousness. This prompted me to experiment with it. I concluded that if it was a good restorative, it might be a useful preventative.

I soon had a chance to test it. A lady called to have an ulcerated tooth extracted. She was in delicate health: face swollen, hands cold. She said she would like to take chloroform, but her physician said she must not take it; she knew she would faint without it. I told her I thought that could be prevented. I took a doily, folded it small, and poured about a teaspoonful of the essence on it. I told her to inhale through her nose, and exhale through her mouth. She continued this until her brain was pretty well stimulated, and the tooth extracted. She showed no signs of syncopy, and could hold a glass of water as still as I could. I have not had any one to faint away in my office since.

In the Spring of 1874, a lady, aged about sixty, came to consult me. She said that her teeth were so bad she could not eat any ordinary food; had disease of the lungs; was forbidden to take chloroform. After an examination, I told her she had eight teeth in the upper jaw that could not possibly be made useful, and she had better have them extracted. She said she had never had one extracted without fainting dead away. She could not think of having more than one out at a time. Her temperament, nervous sanguine, emaciated hands cold, pulse very feeble. I told her if she would follow my directions I would take them all out and she would not mind it more than one, and guaranteed she could not faint if she tried. I explained the effect of the wintergreen, and said it would do no more harm than a glass of good wine. I administered the article, as before described, until her face flushed, tears ran down her cheeks, then extracted the eigth teeth without her closing her mouth. She asked if they were all out, and I said yes. She said, "Is it possible?" I gave her a glass of water. It did not show the least tremor of the nerves. She left, giving me many thanks, saying that she felt much better than when she came into the office.

I could relate many more similar cases, but do not deem it necessary. My object is to show what may sometimes be done to advantage in cases of syncopy, and also the means of preventing it while performing a painful operation.

As before said, the brain is the seat of all sensation; and our patients, no matter how nervous they are, if the brain is properly stimulated, cannot faint—caused by the extraction of teeth. When my patients are known to be pregnant, I always use the stimulant above describcd before performing any painful operation; it always prevents any severe shock of the nervous system when in this condition. If any one wishes to know how to prepare the Essence, it is as follows: To one pint of alcohol add one ounce of the Oil of Gaultheria, commonly called Wintergreen. Shake it well and it is fit for use.

In passing from a conscious to an unconscious state, all the Clairvoyants I have questioned on the subject say it is affected by a change of polarity of the sensatory organs, and the principle is the same whether caused by animal magnetism, syncopy or anæsthetic agents, and if only the voluntary organs are affected thereby, there is no danger to life, but if polarity in the involuntary is reversed, the heart ceases to beat and death is instantly the result. I was the first in this city to administer ether for the purpose of extracting teeth without pain. In a few cases it developed paroxysms of hysteria; otherwise no harm was done. I have administered ether, chloroform and gas to over two thousand persons. With chloroform, I had three cases that barely escaped death in my chair; with gas, some after deleterious effects followed in two cases.

Admitting man's physical organization to be a magnetic machine, the deaths that have occurred are easily explained, when caused by these powerful drugs. The voluntary organs are under the control of the will, and during our waking hours there is a constant draft on our magnetic supply; it is best recuperated by sleep, when the will is at rest.

The involuntary organs do not sleep until death ends our earth life. We con readily understand that if by any cause polarity in the two large poles in the cerebrum are reversed, the gateway by which we gain a knowledge of things about us is closed so perfectly that physical sensation is impossible. On the other hand, if the equilibrium between the two large poles in the cerebellum are not well balanced, just in this proportion some kind of ailment is the result. Let us not forget that the will has no control over these poles, and all medicine that does not benefically act on them is non-curative. Now, just in proportion as anæsthetic agents disturb their equilibrium, they are dangerous, it makes no difference whether polarity is reversed or destroyed; in either case the principle of life can no longer act on the nerves by means of its intermediate; the heart ceases to beat, and restoration is impossible. Chloroform is more easily administered than ether or gas, and most convenient when the patient cannot come to the office; but we should remember that many deaths have occurred when given for the purpose of extracting teeth, and that, too, when least expected. The public mind is more horrified at one death in the dentist's office than twenty caused by a railroad smash-up. We have now the means that will stimulate the nerves, greatly mitigate the pain and not endanger either life or health.

I have long desired a perfectly safe anæsthetic that can be administered no matter what the condition of the patient. I am now creditably informed that Dr. Mayo, of Boston, some eighteen months ago, by various experiments. produced a compound article that satisfied him was harmless. He would not put it on the market until it had been thoroughly tested by both dentists and surgeons. All who tested its effect and efficiency testified to its great superiority over all other known anæsthetics for dental and minor surgical operations. It is now only a few months since he made arrangements for its manufacture and appliances, and put it on the market. He has named it Mayo's Vegetable Vapor Anæsthetic. I have been using it for extracting teeth very successfully. The nitrous oxide causes the patient, when fully under its influence, to have very like the appearance of a corpse. The action of this new anæsthetic does not act on the vital organs, and the patient appears like one in a natural sleep, and, in my opinion, is perfectly safe and without danger to life or health.

Our patients come to us for either a preventative or curative treatment. As before said, we are brought face to face with almost every conceivable condition of the nervous system, and the more true knowlege we have of it the better are we able to satisfactorily manage them. Some come in a very excitable, and some in a very depressed state. We need means to quiet the former and stimulate the latter.

Again. To be fully entitled to the name of Scientific, we must know something of the laws of life, in order that we may obey them and fight life's battles manfully—doing justice to others and with credit to ourselves. Life, in itself, is not creatable, but given to us with power to properly use or abuse, the end being the creation of the finite mind. I have explained above the means by which it acts on matter, but as a further illustration, let me say, you drop an article on the floor, gravitation holds it there; you desire to pick it up, how can you do it; if you have sufficient will-power it will act on the magnetic element, this on the nerves, these on the muscles. You stoop down and pick it up, and probably not one in ten thousand have a single thought about the necessary means by which you are enabled to do so, so little do we reflect about causative principles involved in what we do.

So far as our voluntary organs are concerned they may be compared to a locomotive engine. They are both useless if the motive power is wanting. To make the engine useful, steam must be generated by means of fire and water; and to make our voluntary organs useful, animal magnetism must be generated by means of life and the atmospheres. The engineer controls the steam power, and human will controls the magnetic power, and when properly applied, if the machine is in good order, locomotion is the result in both cases. I will only add, the steam acts on the piston heads and causes the crank to move and the wheels to rotate. Magnetism acts on the nerves, then on the muscles, and man moves in any direction he chooses. A dentist with a strong will, if he uses the proper means, can more easily and favorably impress his nervous patient than one with a weak will, and the reason is, he imparts more of his animal magnetism, which has a stimulating effect on the nerves of his patient. There is a magnetic sphere emanating from both man and beast, particularly when in motion. Were it not so, no dog could follow their tracks successfully. All pain is the result of an obstruction of a normal flow of the magnetic current, whether caused by disease or otherwise.

Arsenic, applied to the nerve of a tooth, destroys its polarity, and applied to any other nerve it is no longer capable of being actuated by the magnetic current, without which there can be no sensation, and death is the result. Physical endurance depends largely on the mind and the state of the nervous system. The difference in individuals to bear pain is marvelous; some one can have a tooth extracted and seem to care but little about it, while others, without the use of a preventative, appear to suffer intensely, and in some cases the operation causes syncopy.

In conclusion allow me to say, a vast field lies before us, and if cultivated properly this Society will in due time reap a rich harvest, the benefits of which cannot now be estimated.

The grand distinction between mind and matter may be seen thus: If we give any physical object to another, we part with it, but if we give a new idea on any subject, we do not part with it, but in so doing its boundaries are enlarged in our minds.

The space occupied in briefly presenting my views on the subject I have chosen is greater than I at first intended, but the fundamental principles which underlie everything with which we have to do, and the importance of fully understanding them, is my only apology for occupying so much of your time.—*Dental Luminary.*

